# Avoiding Academic Burnout: Academic Factors That Enhance University Student Engagement

**DOI:** 10.3390/bs13120989

**Published:** 2023-11-30

**Authors:** Salvador Reyes-de-Cózar, Alba Merino-Cajaraville, María Rosa Salguero-Pazos

**Affiliations:** Department of Communication and Education, Universidad Loyola Andalucía, 41704 Dos Hermanas, Spain; amerino@uloyola.es (A.M.-C.); mrsalguero@uloyola.es (M.R.S.-P.)

**Keywords:** burnout, dropout, academic engagement, higher education

## Abstract

Burnout is one of the major problems in higher education and is linked to a decline in students’ academic performance and achievement. Burnout, when prolonged over time and added to stress and high workloads, promotes the intention to drop out of studies, which translates into negative consequences for individuals and groups. Academic engagement is proposed as an effective alternative to offer solutions to improve the quality of education and counteract current negative trends. This study is based on a correlational–descriptive research design. It aimed to find out to what extent students feel engaged in their university studies and to identify and analyze possible correlations between engagement and specific classroom variables. To this end, a sample of 764 college students was studied. The result showed that students feel connected to and interested in their studies and that the area of knowledge impacts student engagement. They also indicate how learning strategies used in the classroom positively impact academic engagement.

## 1. Introduction

University education is a line of research with a long tradition that aims to provide scientific knowledge to improve teaching in higher education, raise its quality, and achieve better rates of academic success and performance as well as labor market insertion [1,2,3]. Entities such as [4] have collected evidence from key educational indicators, which point out that 10.2% of students drop out of education and training prematurely. In addition to this figure, the Organization for Economic Co-operation and Development (OECD), in its annual report “Education at a Glance 2022. OECD Indicators”, concluded that in higher education, there is a 12% student dropout rate, while only 37% of students finish their degree studies on time [5]. In recent years, these data have been compounded by low motivation rates, a decrease in academic performance, and high levels of anxiety and depression due to burnout among higher education students [6,7,8,9].

Burnout and its academic consequences [10] have become a concern for the scientific community, as burnout is considered one of the major problems in higher education today [11,12,13]. The university is a challenging context in which students are easily susceptible to burnout [14]. Olson et al. [15] pointed out that many students present frequent burnout symptoms. From a more psychological perspective, Norez [16] highlighted its negative impact on mental health and physical well-being. Factors such as moderate exhaustion, cynicism, and feelings of inadequacy are related to burnout in most students [17]. In the educational field, burnout is associated with a decrease in academic performance [18,19], in self-regulation [20], and, consequently, in achievement and in the results obtained [21]. Prolonged burnout added to other factors such as stress or high workload favors the intention to drop out, which translates into negative consequences at both the individual and group levels and for both faculty and students [22]. Likewise, the adverse effects extend beyond the classroom boundaries and are projected into the work environment. Also, burnout among students has been examined [23], and research has highlighted the negative effects on their future job responsibilities. This reality also has an effect at the economic level, as it wastes resources and, consequently, entails various dangers for the growth of talent and social development [24]. Given these effects, research identifying factors associated with university success or failure helps guide proposals that improve the educational quality of university systems to reverse the current data.

Among the solutions to the burnout phenomenon, the scientific community considers engagement to be a critical indicator, both in terms of the quality of university institutions and the teaching quality, in the search for a factor that can improve the current rates [25,26,27,28]. It is beneficial to allocate efforts to improve student engagement in higher education [29], as empirical evidence has shown that students with better academic engagement have lower rates of intention to drop out [9]. In addition, improving engagement is related to better results during a student’s university academic career [29], mitigating the negative effects derived from burnout [30] and improving the percentage of class attendance [31]. In addition to the above, engagement is a crucial indicator for in deepth learning, academic performance, and therefore, success in studies [32,33], harnessing students’ intrinsic potential [34].

In recent years, there has been an effort by the scientific community to conceptualize the construct of academic engagement [35,36,37]. In general terms, its concept is defined as a student’s time investment, quality of participation, and connection with the university and university-related activities [38]. However, the diversity of ways of understanding engagement sometimes makes research difficult, as it affects the delimitation of dimensions that are part of the construct and, consequently, its empirical investigation. However, this need to clarify the concept has led to interesting theoretical advances that have materialized in the identification of different types of engagement. The authors of [39], on the basis of their previous work [40], proposed three types of engagement: attitudinal, emotional, and cognitive. Attitudinal engagement is related to involvement in academic, social, and extracurricular tasks; emotional engagement is linked to students’ positive responses related to educational institutions, their learning, and interactions between teachers and peers. Finally, cognitive engagement relates to scenarios in which students can develop strategies for the self-management of their learning. Redmon et al. [41] used the proposal of these authors by adding two more types of engagement: social and collaborative. In parallel, a multidimensional model posited six pathways through which students can feel engaged with the university: teaching, learning, research, community, other students, and faculty [42]. Alonso-Tapia et al. [43] used the concept of engagement as an element that involves four types of involvement: behavioral engagement, manifested through positive behavior, dedication, and attention to tasks and participation in extracurricular activities; emotional engagement, manifested through reactions to subjects or activities; cognitive engagement, related to the efforts put into the learning process; and finally, agency, related to the student’s interaction through questions and comments during class.

Many authors have proposed this construct, to make up a broad and complex field of study. Although this research did not aim to systematize the literature, two main approaches helped place the proposed contribution: the psychological and pedagogical approaches [44]. In the former, the study of psychological dimensions associated with engagement prevails, focusing on personal or subjective aspects, such as students’ emotional state, motivations, and initial values, or cognitive aspects, such as learning management strategies [45,46,47].

From the pedagogical perspective, the focus is on the educational processes that are generated in learning contexts. This approach considers the extent to which educational institutions can develop or provide resources and strategies that enhance engagement by implementing intervention proposals in education [48,49]. From the latter view, engagement is considered one of the main predictors of learning and academic success. Some researchers [50,51] have concluded that students with higher levels of engagement achieve better academic results.

Therefore, from the pedagogical approach, engagement is critical in predicting both good learning and students’ academic success and potential results [52,53]. In this sense, many authors emphasize that it is a fundamental factor in understanding, improving, and predicting students’ academic and emotional trajectories [35,54]. The authors point out that when students have high levels of engagement, this positively impacts increased attention in class, greater ability to concentrate on studying, more significant activity and participation in the educational community, and a higher level of performance, among others [55,56]. The development and evolution of this approach have led to the relevance of identifying measurable academic factors in the specific classroom context associated with increased engagement in educational contexts.

Numerous studies have focused on identifying dimensions, variables, or educational factors linked to classroom scenarios that may impact engagement. Windham [57] suggested that it is necessary to include elements of interaction and exploration in teaching activities and ensure the relevance of what is to be learned while proposing intellectual challenges to improve students’ involvement in their learning. During the last two decades, the production on this topic has been gaining relevance and has increased significantly, deepening these aspects and highlighting the need to generate empirical scientific studies focusing on teaching–learning interactions in specific classroom contexts [58,59,60,61,62,63,64].

As with conceptualization, the instruments for measuring academic engagement are many and vary in the approach used. Among the most used is the UWES [65], a tool with 17 items classified into three scales: vigor, dedication, and absorption. The vigor subscale consists of six items, the dedication subscale consists of five items, and the absorption subscale consists of six items. The item scores range from 0 (never) to 6 (always), allowing both total and subscale scores to be found. Wang [66] proposed The Assessment of School Engagement, which assesses this construct through 23 items on a Likert-type scale (1 = rarely and 5 = almost always), distinguishing between emotional (8 items), attitudinal (7 items), and cognitive (8 items) engagement. Shernoff et al. [67] provide information on students and institutions with the NSSE scale, consisting of five categories: academic challenges, active and collaborative learning, institution–student interaction, enriching learning experiences, and support from the university context. This scale is oriented towards higher education and creating proposals for improvement. In turn, the HSSSE [68], the most widely used scale in the United States, aims to investigate students’ attitudes and perceptions, as well as their beliefs about their studies, their learning context, and their interaction with the university community through three dimensions: psychological, emotional, and life engagement. The scale aims to study the relationships that shape students’ experience in university institutions, such as the relationships between the student community and the school, adults and students in the school, students and peers, students and instruction, and students and curricula. The Multifactorial Mixed Scale of Educational Engagement (MMSEE) [44] stands out among recent engagement measurement tools. The MMSEE comprises 34 items divided into five dimensions (motivations, values, learning contexts, emotional state, and management strategies). One of the main characteristics of this scale is, on the one hand, that it aims to bring together dimensions of both approaches (psychological and pedagogical) and, on the other hand, that the dimension “Classroom variables” focuses specifically on observing specific classroom educational scenarios. This paper uses the MMSEE and, specifically, the dimension “Classroom variables” for its study. This scale, in turn, is divided into four factors (relevance, exploration, intellectual challenges, and interaction) which are described below:

Relevance, according to Freitas and Almeida [69], is considered one of the fundamental aspects of generating engagement. Relevant content is related to one’s current interest, contributes to one’s future goals, and is considered significant in shaping one’s identity. In this sense, the authors link this sub-dimension to the promotion of content that is connected to aspects of interest to students. Providing relevant and engaging teaching that relates content to real life is now more critical than ever [70]. Connecting to the real world allows students to ask questions and investigate problems relevant to them [71], thus increasing their engagement.

Exploration creates different scenarios in which the learner can address different practical problems by developing their capacity for autonomy and independence [72,73]. The learning environment should allow the learner to explore and broaden their understanding of the issues they are interested in. The questions they explore should be simple but authentic, requiring real answers [74]. In this sense, it becomes necessary to offer self-directed and interactive activities that allow students to access numerous sources of information and other resources that favor creating and developing meaningful learning [75,76]. Moreover, today’s students are explorers by nature, excelling as autonomous learners through video tutorials or trial and error. Therefore, curricula, methods, strategies, and activities must be attuned to today’s learners’ potential characteristics [77].

Intellectual challenges are essential for fostering engagement in the classroom, as indicated by numerous researchers [78,79,80,81]. Incorporating intellectual challenges in university education implies the creation of contexts that facilitate and develop students’ natural interest in learning so that the teacher mediates their learning by acting as a guide.

Finally, interaction is also considered one of the most relevant elements for generating engagement in the classroom [82]. Kanelopoulos et al. [83] conceive interaction as an engine driving teaching–learning processes and highlight, like Dao and McDonough [84], the relevance of the construction of learning through social interaction. Vygotsky [85] created the theoretical basis for the relevance of social interaction in learning processes. He considers learning to be an interactive process of a social nature, in which there is a transfer between different agents, with language and other cultural elements being the main mediating tools and situated in a specific context or environment that is socially and historically influenced. From a pedagogical approach, engagement is also derived from the reciprocal interaction between students and learning contexts with an eminently interactive and dialogical component [86].

The studies presented highlight how academic engagement in the classroom is nurtured and promoted through various factors: the relevance of the content being taught, the promotion of an exploratory environment, the presentation of intellectual challenges, and the facilitation of meaningful interactions. Each component plays a different role in how students connect, participate, and engage in their learning process. The convergence of these dimensions offers a complete picture of how the classroom environment, pedagogical strategies, and interactions can be designed and aligned to maximize students’ engagement. Given the need to understand these aspects in depth and in a contextualized manner, it becomes crucial to formulate specific questions to guide the exploration of the relationship between these factors and the level of academic engagement in classroom educational contexts. For this purpose, the following research questions were proposed for this study.

RQ1: To what extent do students feel connected and engaged in university studies?
RQ2: Do students’ perceived engagements differ according to subject areas?
RQ3: Is there a relationship between the classroom variables studied and the level of engagement perceived by students?
RQ4: What are the specific classroom variables that most influence the engagement of university students?

## 2. Materials and Methods

### 2.1. Design

This study was conducted according to the Ethical Principles of Psychologists and Code of Conduct from the American Psychological Association (APA). Prior to the research, all participants signed an informed consent. This paper is based on a correlational-descriptive research design. This methodology was selected to describe and document specific characteristics of academic engagement and identify and analyze possible correlations between the selected variables. Not manipulating the variables allows us to observe them as they manifest themselves in their natural environment. It is essential to underline that although the descriptive–correlational design may reveal trends and associations between variables, it does not establish causal relationships. Therefore, the conclusions derived from this study focus on highlighting observed relationships and patterns without inferring direct causes. This design ensures an objective and non-intrusive approach to the study phenomenon, providing an authentic and unaltered picture of the variables and their interaction.

### 2.2. Sampling

The sample participating in this study consists of 764 higher education students (University of Seville, Spain). Following the simple random sampling criterion for finite samples, this sample is statistically significant with a confidence level of 99%, assuming an error of 4.6% for a total population of approximately 60,000 enrolled students. In addition, stratified and quota selection was conducted to obtain a sample that is as representative as possible.

### 2.3. Instruments

For this study, we used the MMSEE instrument, a reliable instrument validated by CFA [44]. As the Introduction Section mentions, this instrument consists of 34 items divided into five dimensions (motivations, values, classroom variables, emotional state, and management strategies). For this study, we used the dimension “Classroom variables”, which focuses on analyzing the classroom environment, pedagogical strategies, and how interactions can be designed and aligned to maximize engagement. This dimension is further divided into four factors (relevance, exploration, intellectual challenges, and interaction, described in the Introduction Section). The scale consists of 17 items (Likert-type response from 1 to 5, with 1 being the minimum and 5 the maximum) that ask how the presence or absence of these academic classroom factors could improve their levels of engagement. Specifically, they are asked, “I get more involved in my studies when…”. In addition, a variable was added to collect this information to measure the overall level of engagement perceived by the students. In this item, students were asked to rate their perceived level of engagement with their studies on a scale of 1 to 5.

Therefore, the final instrument consists of 18 items. Data were collected on paper in the different faculties to ensure that all students understood the items. Throughout the process, the students were accompanied by a researcher to clarify and resolve any doubts regarding their understanding of the items.

### 2.4. Data Analysis

Both descriptive and correlational techniques were used for data analysis. In descriptive terms, we used frequencies, measures of central tendency, and dispersion: mean, maximum, minimum, standard deviation, and variance. These statistics provide an overview of the patterns observed in the sample. Regarding correlational analysis, Pearson’s correlation coefficient was used to determine the linear relationship between the study variables. This technique makes it possible to identify and quantify the strength and direction of the relationships between variables. All calculations and statistical analyses were conducted using SPSS software, version 24.

## 3. Results

### 3.1. Sample Characteristics

Stratified selection by quotas was made to obtain as representative a sample as possible. The stratification criteria used are areas of knowledge and academic year levels. This stratification is reflected in the composition of the sample obtained. Thus, 20.4% of the sample is made up of students in the first year of the degree, 20.4% corresponds to students in the second year, 20.9% to the third year, 20.4% to the fourth year, and 17.9% to the Master’s/Postgraduate degree. Regarding areas of knowledge, 19.9% represent Health, 20.9% Social Sciences, 20.7% Engineering and Architecture, 17.8% Science, and 20.7% Arts and Humanities. The average age of the university students surveyed was 22 years, with an SD = 4.029. As for the student’s gender, parity was sought in the areas of knowledge and the courses, resulting in a final distribution of 50.5% men and 49.5% women.

The distribution of the groups according to courses and areas of knowledge is shown below. As shown in Table 1, the proportions of the groups are as homogeneous as possible.

### 3.2. Internal Structure of the Instrument

Although the MMSEE is a reliable instrument and validated by CFA [44], a principal component analysis (PCA) was carried out in this study to explore the validity of the scale’s internal structure. The data obtained from the KMO (*p* = 0.913) and Barlett’s test of sphericity (*p* = 0.0005) allow us to conclude that the PCA was relevant. A Promax oblique rotation with Kaiser normalization was used to gain more interpretability.

As seen (Table 2), the resulting factorial solution comprises four factors capable of jointly explaining 62.58% of the total variance. On the other hand, the scale presents a high index of overall internal consistency (α = 0.865) and satisfaction by factors, considering the number of items per factor and the purpose of the questionnaire [87,88].

### 3.3. Level of Engagement Perceived by University Students

**Figure 1:**
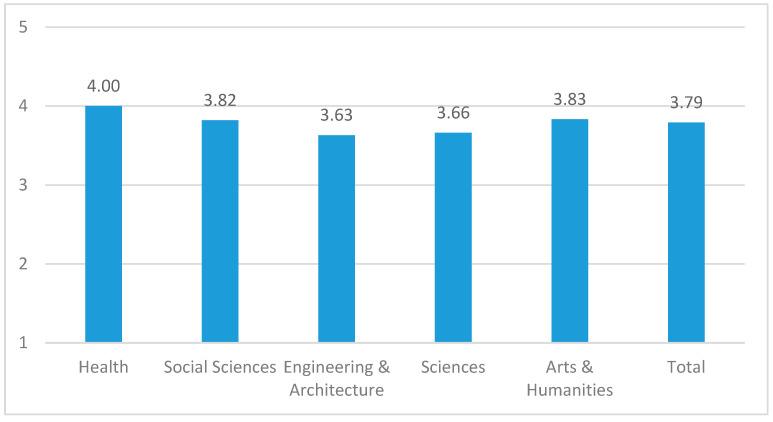
Average global engagement level perceived by students: disaggregated by areas of knowledge and overall average.

Concerning RQ1 and RQ2 of the study, which aims to find out to what extent students feel connected to their university studies and whether the level of engagement students perceive differs according to subject areas, Figure 1 shows the results broken down by subject area. In general terms, the results show that the engagement level with their studies is medium-high, with an overall average of 3.79 out of 5.

Looking at the different areas of knowledge, it is worth noting that Health (4) and, to a lesser extent, Arts and Humanities (3.83) and Social Sciences (3.83) stand out, being above the overall average. On the other hand, the lowest values are found in the areas of Science (3.66) and Engineering and Architecture (3.63), both within the “experimental sciences” group.

**Figure 2:**
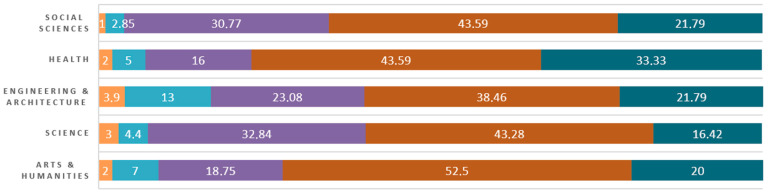
Engagement levels by areas of knowledge in percent.

Looking at the individual percentages (Figure 2), the low number of students who consider themselves with a low level of engagement is remarkable. Thus, less than 20% of the students are concentrated in the values a little or not at all in the five areas. On the other hand, more than 60% of students consider themselves quiet and very engaged concerning their level of connection with their studies. However, despite this data, only in the field of Health does the level very much represent a percentage close to 40%, and in the rest of the areas, approximately 20%. In general, there is a tendency for the highest percentage of students in the five areas to be concentrated on the value of quite a lot. Thus, the data obtained indicates that university students’ perceived level of engagement is medium-high.

### 3.4. Relationship between the Level of Engagement and Classroom Variables

To answer RQ3 of the study, correlational analysis techniques were applied to explore whether there is a relationship between the classroom variables studied and the level of engagement perceived by the students. As can be seen in Table 3, all the proposed variables show a statistically significant correlation (*p* = 0.001) with the variable level of engagement, and it can be affirmed that there is a directly proportional relationship between the levels of engagement perceived by the students and the different classroom variables studied.

### 3.5. Classroom Factors Associated with Improved Engagement

Finally, and concerning RQ4 of the study, which focuses on identifying which specific classroom variables have the most significant influence on the engagement of university students, the four factors studied show medium-high average values. These data indicate that, according to the students surveyed, academic contexts, where these characteristics are reproduced efficiently and continuously, would improve their level of engagement. Specifically, the Interaction factor is the highest rated (3.95 out of 5). In second place is Exploration (3.85), followed in third and fourth place by Intellectual Challenges (3.41) and Relevance (3.28).

As shown (Table 4), Interaction receives the highest score in the study (3.95). Students are well-regarded in improving their engagement by feeling part of a team, expressing their opinions, and having good communication with peers and teachers.

For the Exploration factor (Table 5), the item “I find a positive attitude on the part of my tutors and teachers to attend to my needs” obtained a score of 3.94, followed by “I find the teachers’ explanations stimulating,” with an average value of 3.91. Compared to these items are others with lower values, such as “I find the teachers’ explanations easy to understand and connected to my interests” (3.70).

Intellectual challenges (Table 6) are one of the factors least valued by the students. It is the most highly rated item with 3.51 “The activities require the maximum of me to overcome them”. In contrast, the item “In class, I use all the possibilities of the new technologies” obtains the lowest value within this factor (3.22).

Finally, the Relevance factor (Table 7) is the one that obtains the lowest values, including “The teachers use the doubts I raise in class to broaden the content of the subjects” (3.14) and “My teachers invite professionals from the world of work to the classes” (3).

Based on the data presented, it can be deduced that university students’ engagement is significantly influenced by certain intra-classroom variables, with Interaction as the pre-eminent factor. Educational institutions should reflect on these findings, especially about the less valued aspects such as Relevance, to optimize and strengthen the academic environment towards greater student participation and engagement.

## 4. Discussion

First, using the MMSEE to assess university students’ academic engagement levels provides a scale applicable to other studies on engagement in university classrooms. Although there are already studies that measure levels of engagement, most of these are limited to recording the construct from strictly psychological dimensions. This scale is of great pedagogical interest in university settings as fewer studies address this construct from a pedagogical perspective, and even fewer do so from a mixed perspective. Adopting mixed methodologies, with psychological and pedagogical approaches, provides a deeper understanding of engagement, allowing the complexity inherent in this phenomenon to be accurately addressed.

When students are asked as to what extent they are connected and engaged with their studies, the data reveal that, although a large percentage feel motivated, there is still a significant proportion of students (around 20–40%, depending on the area of knowledge) who claim to be some, a little, or not at all engaged with their university studies. This demotivation leads to an increased risk of burnout and, consequently, of dropout, a serious university problem affecting education internationally [89,90,91,92,93]. These findings converge with research by several authors in this field and with statistics published by official bodies, which point out that seven out of ten students experience or are at risk of burnout [94] directly related to dropping out of school. For example, in the United States, the dropout rate is around 30% [95], while in countries such as Spain, this rate reaches 12% [5]. In this line, burnout and dropout hurt students and the education system, implying a decrease in self-efficacy [96], a decrease in students’ well-being and academic performance [97], and, therefore, poorer achievement and outcomes [21]. Among the solutions to address this reality, many authors consider engagement a critical strategy, as it is essential in reducing academic burnout among students [98,99,100]. Universities need to maintain a desirable level of student engagement, from general and institutional [101,102,103] to academic and classroom solutions [89].

On the other hand, looking at the values of engagement students perceive according to areas of knowledge, two large groups can be defined. Thus, one group would be formed by Health, Social Sciences, and Arts and Humanities, obtaining the highest values, and the other group, with lower averages, by Sciences, Engineering, and Architecture. This finding coincides with the study conducted by Fernández-García et al. [104], in which students evaluated the effectiveness of teachers in the areas of Arts and Humanities, Social and Legal Sciences, and Health Sciences more positively. These results could reveal how a more humanistic dimension is associated with engagement, while content aimed at abstraction or science generates low engagement. Authors such as [105] highlight the lack of application of active learning in science degrees, where most students are unfamiliar with these teaching strategies [106]. Other research [107] adds that traditional educational practices are beginning to be inappropriate for engineering students, gradually incorporating active learning strategies. Several authors highlight how using active learning strategies in engineering favors better results than traditional methodologies [107,108]. Along the same lines, Sukkar et al. [109] point out an improvement in the motivation and participation of architecture students thanks to the implementation of active teaching techniques. This data corroborates that the teaching–learning strategies used in the classroom play a fundamental role in student performance, showing a direct relationship with engagement levels [104,110,111] and how implementing these strategies in the classroom leads to better student satisfaction and performance data [112]. Numerous studies highlight the positive impact of student-centered learning strategies such as flipped classrooms, problem-solving, and case studies, among others, improving students’ class participation and cognitive attention [113,114], a perception teachers and students share [115]. These studies are of great value from an applied point of view, as they provide scientific support for the need to orient university teaching in the classroom towards didactic methodologies that enhance specific elements that generate engagement.

Therefore, modifying teaching styles and identifying classroom factors that enhance engagement are of interest in this path of improvement in the teaching–learning processes in higher education. In this sense, the discovery of dimensions associated with classroom work that strengthen student engagement opens up a possible path that can contribute to the inclusion of this group. In line with RQ3, this study analyses factors that can improve university students’ engagement levels, finding a statistically supported positive and significant correlation between these established dimensions and engagement. From an applied perspective, the results of this work support the importance of teachers adopting strategies and ways of working in the classroom that include the factors analyzed in this study. These factors strengthen engagement, as positive experiences in learning environments and are associated with greater academic engagement and decreased burnout [8].

The dimensions explored in this study that impact the level of engagement are Interaction, Exploration, Intellectual Challenges, and Relevance. The main factor with the best results is Interaction in the involvement of university students in their learning, with fluid communication with classmates and teachers standing out as the most highly valued item. This dimension can be translated into incorporating interactive, collaborative, and teamwork methodologies in the classroom to increase student involvement in their studies. The results obtained converge with other research carried out in other university contexts. Thus, Gutiérrez et al. [116] concluded that an incentive for student autonomy on the part of the teacher leads to more significant commitment, participation, or engagement. Other studies also converge on the same idea [117] and relate the need to apply group work techniques in the classroom with students as a mechanism to improve their interaction [118], also pointing out how motivation and academic performance data improve due to the positive impact of techniques that promote student participation and collaboration [119,120].

On the other hand, students’ second most valued factor is Exploration. According to Vygotsky [85], exploration is related to intellectual curiosity and the investigation of spaces of proximal development provided by the teacher, thus reflecting the teacher’s role in this dimension. Students expect their teacher’s role to be a mediator between knowledge and students’ interests, to stimulate curiosity, and to be a personal support in their development, as the item with the highest values is the one that mentions the importance of finding a positive attitude towards understanding their needs on the part of tutors and teachers. Vracheva et al. [121] show how developing students’ curiosity improves their engagement figures. Asmin [122] also highlights the importance of fostering students’ curiosity to enhance creativity and motivation. Another study [123] emphasizes the importance of teachers paying particular attention to curiosity by designing practices and sessions that promote and encourage it.

The third factor, Intellectual Challenges, leads us to consider the relevance of incorporating educational models closer to the new generations’ learning styles, such as using technologies and incorporating activities that challenge the students’ intellectual capacities. This fact is especially crucial as we are beginning a new technological era led by generative artificial intelligence and commercial and educational tools specialized in creating images, text, and other content. The findings coincide with others, such as the study by Rigo et al. [124], in which students, with 96% positive ratings, highlight the need for innovation in the classroom and the need to face challenging classroom contexts. Similarly, some studies [125,126] highlight the positive attitudes of university students toward the use of technology in education and how most of them state that interactive educational technologies positively affect their learning abilities as they are a challenge to them and, at the same time, awaken their curiosity and peer interaction. Other studies, such as Chu et al. [127] and Paganini et al. [128], point to mobile applications as crucial tools for reducing university students’ stress and burnout levels and improving their attention and well-being.

Furthermore, finally, Relevance highlights the importance of connecting with students’ prior knowledge and interests and giving contextualized meaning to disciplinary knowledge. To this end, Garcia and Pintrich [129] mention the need for teachers to understand students’ prior knowledge to integrate motivational and cognitive components that regulate motivation, cognition, and learning in the classroom. Similarly, several studies point out how teaching relevant content aligned with students’ interests impacts student engagement, improving student interest, increasing the perceived value of the task, and thus improving their academic outcomes [130,131]. Arnold [132] also showed that courses that focused on addressing students’ needs and interests and improving their connection to their studies prepared them better for the world of work. Similarly, Floris et al. [133] showed how career-focused planning helps regulate students’ efforts by increasing their confidence, self-regulating their learning, and improving their academic results. Therefore, teaching that incorporates relevance and connects with students’ interests will improve university students’ academic outcomes.

This study empirically contrasts pedagogical dimensions associated with university engagement. Although the study of engagement relationships with university performance occupies the attention of numerous current studies, the study of variables such as subjective well-being, motivation, satisfaction, feelings of self-efficacy, and learning [134] prevails in these studies, in which academic engagement is still addressed as a psychological problem [24]. Therefore, there are remaining studies that still need to incorporate connections between pedagogical variables and engagement. In this line, Nyklová and Aleja [135] proposed research with a more pedagogical approach to the construct due to the lack of studies that address it from this point of view. In this sense, the data obtained in this study are a step forward in developing this line of research.

These results provide information for creating educational models that improve university success rates and reduce academic dropout. Therefore, this work may be of scientific interest as it provides empirical data on the relationship between academic classroom factors and engagement in the university environment, a context in which there are few or no studies [136], hence the need for further research in this direction. Therefore, it may constitute a starting point for future research interested in exploring factors that affect the performance and success of their students, trying to shed light on aspects that enhance engagement as a critical factor in university performance and success.

## 5. Conclusions

The results show students are connected to and interested in their studies, as opposed to other theories that describe populations of young people who have little or no interest in or see the value for studying. Likewise, these data confirm that some students feel disengaged from their studies, which translates into a potential risk of dropping out. Therefore, knowing the factors within the classroom that can help teachers improve all students’ engagement levels is a step toward in understanding engagement and reducing burnout.

The subject areas in which the grades are framed directly impact student engagement. These degrees’ teaching and learning strategies can be linked to this fact. Active learning and other student-centered teaching strategies are more common in social sciences and other humanistic fields than in other fields. In the experimental sciences, these methods are rare, impacting their students’ engagement.

The classroom variables studied, Interaction, Exploration, Intellectual Challenges, and Relevance, correlate with student engagement. This data implies that it is possible to obtain educational spaces that favor student engagement by introducing, modifying, or modulating these variables in the classroom. This information allows teachers, on the one hand, to evaluate the type of teaching they carry out and improve those aspects that do not generate engagement and, on the other hand, to improve the classroom climate through simple strategies that are easy to apply and measure.

Interaction as a factor in the classroom is based on generating learning environments where communication is encouraged through group work techniques. This fact positively impacts students as it improves their motivation and involvement, improving academic performance. Exploration is related to the development of students’ curiosity and creativity. When the classroom allows space for students to explore and develop their curiosity, their motivation and, therefore, their engagement levels are positively affected. The learning style of the new generation demands intellectual challenges in the classroom. Students positively value the use of technological resources and virtual environments, thus causing them to be more connected to their peers. This fact translates into students with lower levels of burnout or demotivation or, in other words, higher levels of engagement. Teaching content in tune with students’ interests and prior knowledge is reflected in better academic results, increasing students’ motivation and interest in learning. Therefore, in an environment where students’ interests are considered, and relevant content is taught, students’ connection to their studies will improve, thus avoiding the possible dropout risk. However, for these results to be successful and implemented by all teachers, institutions must be involved at the same level as teachers. Too often, higher education institutions focus on research incentives, neglecting crucial aspects such as the quality of teaching and the well-being of students.

The MMSEE [44] is a valid and reliable instrument that allows us to measure engagement from a mixed perspective. Specifically, in this study, we used the dimension of classroom variables that focus on measuring pedagogical variables implemented in classroom educational contexts. Using mixed instruments (psychological-pedagogical) allows for a better understanding of engagement and offers specific solutions that respond to the phenomenon’s complexity.

The results obtained in this study serve as a basis for improving university success rates. Obtaining educational spaces where students’ perceived engagement with their learning is favored translates into improved performance, high motivational levels, and a lower risk of academic dropout.

## Figures and Tables

**Table 1:** Distribution of the sample by year and area of knowledge.

		n = 764
		Percentage (%)
Course	First	20.4
	Second	20.4
	Third	20.9
	Fourth	20.4
	Master’s/Postgraduate	17.9
Areas of knowledge	Science	17.8
	Social Sciences	20.9
	Health	19.9
	Engineering and Architecture	20.7
	Arts and Humanities	20.7

**Table 2:** PCA solution of classroom engagement factors and reliability coefficients.

PCA			Reliability
Factors	No. of Items	% Variance	Cronbach’s α
Relevance	6	32.625	0.847
Exploration	4	12.997	0.793
Intellectual challenges	4	9.516	0.815
Interaction	3	7.447	0.759
Summary (n = 764)	17	62.585	0.865

**Table 3:** Results of correlational analysis of classroom variables with perceived global engagement.

Variables	Engagement	
	Coef.	Sig.
Relevance	0.133 **	0.010
Exploration	0.137 **	0.008
Intellectual challenges	0.140 **	0.007
Interaction	0.209 **	0.000

** *p* = 0.001.

**Table 4:** Descriptive statistics on classroom factors: Interaction.

	I Am More Involved in My Studies When…/I Have More Engagement in My Studies When…					
Factors	Item	$\bar{x}$	Min.	Max.	s	s2
Interaction	I feel integrated and part of a work team	3.99	1	5	0.94	0.89
	I can express my opinions and discuss them	3.86	1	5	1.01	1.03
	I have fluent interpersonal communication with colleagues and teachers	4	1	5	0.93	0.87
	Total	3.95	1	5	0.76	0.57
	1 = Not at all. 2 = A little. 3 = Some. 4 = Quite a lot. 5 = A lot.					

**Table 5:** Descriptive statistics on classroom factors: Exploration.

	I Am More Involved in My Studies When…/I Have More Engagement in My Studies When…					
Factors	Item	$\bar{x}$	Min.	Max.	s	s2
Exploration	I find the teachers’ explanations easy to understand and connected to my interests	3.70	1	5	1.08	1.18
	I find the teachers’ explanations stimulating	3.91	1	5	1.11	1.24
	In the classes, questions arise that provoke curiosity or the desire to inquire about them	3.88	1	5	0.98	0.96
	I find a positive attitude on the part of my tutors and professors to attend to my needs	3.94	1	5	1.02	1.04
	Total	3.85	1	5	0.84	0.70
	1 = Not at all. 2 = A little. 3 = Some. 4 = Quite a lot. 5 = A lot.					

**Table 6:** Descriptive statistics on classroom factors: Intellectual Challenges.

	I Am More Involved in My Studies When…/I Have More Engagement in My Studies When…					
Factors	Item	$\bar{x}$	Min.	Max.	s	s2
Intellectual Challenges	In class I use all the possibilities of new technologies	3.22	1	5	1.14	1.30
	The professors propose the subjects with activities that require autonomy (research work, voluntary, open topic, etc.)	3.44	1	5	1.10	1.22
	The teachers facilitate the use of different sources or technological resources (audiovisual media, internet, blogs, etc.) for the development of the subjects	3.48	1	5	1.08	1.17
	The activities demand the maximum from me to overcome them	3.51	1	5	0.97	0.95
	Total	3.41	1	5	0.76	0.58
	1 = Not at all. 2 = A little. 3 = Some. 4 = Quite a lot. 5 = A lot.					

**Table 7:** Descriptive statistics on classroom factors: Relevance.

	I Am More Involved in My Studies When…/I Have More Engagement in My Studies When…					
Factors	Item	$\bar{x}$	Min.	Max.	s	s2
Relevance	The teachers use the doubts I raise in class to expand the content of the subjects	3.14	1	5	1.19	1.41
	Teachers find meaning in the subject matter that I consider useful in other contexts	3.28	1	5	0.99	0.98
	The review of exams and evaluation tests helps me to clarify and learn about my mistakes	3.38	1	5	1.12	1.26
	In the classroom I work on activities related to possible work problems	3.23	1	5	1.19	1.43
	The doubts I raise in class are satisfactorily resolved	3.68	1	5	1.04	1.09
	My professors invite professionals from the working world to the classes	3	1	5	1.14	1.30
	Total	3.28	1	5	0.79	0.63
	1 = Not at all. 2 = A little. 3 = Some. 4 = Quite a lot. 5 = A lot.					

## Data Availability

The data presented in this study are available on request from the corresponding author.
